# The role of EORTC QLQ-C15-PAL scores and inflammatory biomarkers in predicting survival in terminally ill patients with cancer

**DOI:** 10.1186/s12885-021-08049-3

**Published:** 2021-03-23

**Authors:** Nanako Koyama, Chikako Matsumura, Yoshihiro Shitashimizu, Morito Sako, Hideo Kurosawa, Takehisa Nomura, Yuki Eguchi, Kazuki Ohba, Yoshitaka Yano

**Affiliations:** 1 Education and Research Center for Clinical Pharmacy, Kyoto Pharmaceutical University, 5-Nakauchi-cho, Misasagi, Yamashina-ku, Kyoto, 607-8414 Japan; 2 Department of Pharmacy, Tachibana Medical Corporation Higashisumiyoshi-Morimoto Hospital, Osaka, Japan; 3 Palliative Care Unit, Tachibana Medical Corporation Higashisumiyoshi-Morimoto Hospital, Osaka, Japan; 4 Department of Palliative Care, Tachibana Medical Corporation Higashisumiyoshi-Morimoto Hospital, Osaka, Japan

**Keywords:** EORTC QLQ-C15-PAL, Inflammatory biomarkers, Survival prediction, Terminally ill cancer patients, Palliative care

## Abstract

**Background:**

The clinical use of patient-reported outcomes as compared to inflammatory biomarkers for predicting cancer survival remains a challenge in palliative care settings. We evaluated the role of the European Organization for Research and Treatment of Cancer Quality of Life Questionnaire Core 15 Palliative scores (EORTC QLQ-C15-PAL) and the inflammatory biomarkers C-reactive protein (CRP), albumin (Alb), and neutrophil-lymphocyte ratio (NLR) for survival prediction in patients with advanced cancer.

**Methods:**

This was an observational study in terminally ill patients with cancer hospitalized in a palliative care unit between June 2018 and December 2019. Patients’ data collected at the time of hospitalization were analyzed. Cox regression was performed to examine significant factors influencing survival. A receiver operating characteristic (ROC) analysis was performed to estimate cut-off values for predicting survival within 3 weeks, and a log-rank test was performed to compare survival curves between groups divided by the cut-off values.

**Results:**

Totally, 130 patients participated in the study. Cox regression suggested that the QLQ-C15-PAL dyspnea and fatigue scores and levels of CRP, Alb, and NLR were significantly associated with survival time, and cut-off values were 66.67, 66.67, 3.0 mg/dL, 2.5 g/dL, and 8.2, respectively. The areas under ROC curves of these variables were 0.6–0.7. There were statistically significant differences in the survival curves between groups categorized using each of these cut-off values (*p* < .05 for all cases).

**Conclusion:**

Our findings suggest that the assessment of not only objective indicators for the systemic inflammatory response but also patient-reported outcomes using EORTC QLQ-C15-PAL is beneficial for the prediction of short-term survival in terminally ill patients with cancer.

**Supplementary Information:**

The online version contains supplementary material available at 10.1186/s12885-021-08049-3.

## Background

For patients with advanced cancer, the accurate prediction of survival is important for clinical and personal decision-making in the last months, weeks, and days of life. In clinical practice, the prognosis is based on various factors including symptoms and biomarker measurements [1, 2]. As the end of life approaches, the various distressing symptoms increase in severity [3], and the most significant include deterioration in performance status, dyspnea, delirium, and cancer cachexia syndrome [4, 5]. Therefore, multidimensional information about not only objective variables such as laboratory values but also subjective variables such as patients’ symptoms play an essential role in the accurate prediction of survival.

A patient-reported outcome (PRO) is a measurement based on a report from a patient without amendment or interpretation by a clinician or anyone else [6]. Some of the scales in PRO have been significantly associated with survival and identified as independent prognostic factors influencing survival in patients with advanced cancer [7–12]. However, despite previous studies supporting the added prognostic value of PROs, their systematic use during the cancer treatment and palliative care process remains unknown [11].

In palliative care settings, quality of life (QOL) is the most important outcome. One of the common instruments for assessing QOL is the European Organization for Research and Treatment of Cancer Quality of Life Questionnaire Core 15 Palliative Care (EORTC QLQ-C15-PAL), which is a shortened version of the EORTC Quality of Life Questionnaire Core 30 (QLQ-C30) [13, 14]. Lee et al. showed that the QLQ-C15-PAL can be an independent prognostic factor in inpatients with cancer near the end of life [8]. However, studies about the accuracy of prognoses estimated by QLQ-C15-PAL scores are limited. In addition, the adequate cut-off values of these scores have not been explored.

Moreover, terminally ill patients with cancer frequently experience symptoms associated with cancer cachexia, which is characterized by weight loss, muscle wasting, fatigue, and appetite loss [15, 16]. Cancer cachexia is considered a metabolic disorder driven by the systemic inflammatory response [17]. The presence of systemic inflammatory response, as evidenced by high C-reactive protein (CRP) [18, 19], low albumin (Alb) [20, 21], and high neutrophil-lymphocyte ratio (NLR) [22–24], also has independent prognostic value in patients with advanced cancer. These inflammatory biomarkers have been shown to be reliable prognostic markers in cancer patients with numerous cancer sites [25]. The modified Glasgow Prognostic Score (mGPS) [26], assessing the magnitude of the systemic inflammatory response, is one of the validated prognostic tools based on objective criteria using a combination of CRP (> 1.0 mg/dL) and Alb levels (< 3.5 g/dL) [25–27]. However, a previous study reported that the prevalence of CRP ≥ 1.0 mg/dL was 79.6% in patients with advanced cancer in palliative care settings [18]. Therefore, the preferred cut-off value for terminally ill patients with cancer needs to be further investigated.

This study aimed to examine the role of QLQ-C15-PAL scores and the inflammatory biomarkers CRP, Alb, and NLR for survival prediction to help avoid discomfort and inappropriate therapies in terminally ill patients with cancer.

## Methods

### Study design

We conducted a prospective observational study of patients with advanced cancer hospitalized to receive palliative care in a palliative care unit from June 2018 to December 2019. The inclusion criteria were (1) consent to participate in the study and (2) the ability to complete the questionnaires. The exclusion criteria were inability to complete the questionnaires due to lack of consciousness or cognitive impairments. All participants provided verbal informed consent to use their data in the study and were followed until death or discharge during the study period. The study was conducted according to the Declaration of Helsinki and the Ethical Guidelines for Epidemiology Research and was approved by the ethics committees at the hospital on May 15, 2018 and the university with which the authors were affiliated. The recruited patients in this study were the same as those included in a previously reported study [28].

### Collection of data regarding clinical parameters

The study was conducted as a part of the routine practice of health care professionals in the palliative care unit. All clinical parameters were measured at the time of patients’ hospitalization in the palliative care unit. Patients’ data, including those for age, sex, primary cancer type, presence of metastatic disease, Palliative Performance Scale scores (PPS), and the inflammatory biomarkers CRP, Alb, and NLR, were collected from their electronic medical records. The NLR was defined as the absolute neutrophil count divided by the absolute lymphocyte count [29]. The mGPS was calculated as follows: mGPS = 0 (CRP ≤ 1.0 mg/dL), 1 (CRP > 1.0 mg/dL and Alb ≥3.5 g/dL), and 2 (CRP > 1.0 mg/dL and Alb < 3.5 g/dL). These cut-off values are based on those used in previous studies examining the mGPS [26].

### Measurements of QOL

Patients’ QOL and clinical symptoms were assessed using the Japanese version of the EORTC QLQ-C15-PAL. Patients completed the questionnaires at the time of hospitalization in the palliative care unit. The QLQ-C15-PAL consists of 15 questions pertaining to QOL and includes 2 multi-item functional scales (physical and emotional functioning), 2 multi-item symptom scales (fatigue and pain), 5 single-item symptom scales (dyspnea, insomnia, appetite loss, constipation, and nausea/vomiting), and a question regarding overall QOL (global health status). Most responses were provided using a 4-point scale (1 = “not at all,” 2 = “a little,” 3 = “quite a bit,” and 4 = “very much”), and overall QOL was rated using a scale ranging from 1 (very poor) to 7 (excellent). All 10 scales were linearly transformed according to a previous publication [13, 30]; these transformed scores ranged from 0 to 100. Higher scores indicate better health-related QOL in the functional scales and the overall QOL scale and worse symptoms in the symptom scales.

### Statistical analysis

Survival time was defined as the period from the date of admission to the palliative care unit to the date of death. Patients without available information regarding survival because they left the palliative care unit or remained in the palliative unit during the study period were considered to be censored. The Cox proportional hazard model was used to evaluate the influence of QLQ-C15-PAL scores and inflammatory biomarkers on survival times. We performed a univariate regression, individually entering the 10 transformed QLQ-C15-PAL scale scores and the inflammatory biomarkers CRP, Alb, and NLR as independent variables into the Cox model. In addition, we performed a multivariate regression, including only statistically significant variables with a *p* value < .05 in the univariate analysis. Missing data were excluded.

The cut-off values for detecting the risk of a short-term prognosis of < 3 weeks (21 days) for the statistically significant factors according to the results of univariate Cox regression were determined via receiver operating characteristic (ROC) analysis for patients for whom survival times were obtained. We selected 3 weeks because this term is clinically important for decision-making in end of life care [31]. We divided patients into 2 groups based on these cut-off values, and the survival curves were drawn using the Kaplan-Meier method, with the log-rank test used to compare survival times between the 2 groups. All analyses were performed using BellCurve® for Excel Version 2.15 (Social Survey Research Information Co., Ltd.), and *p* values < .05 were considered statistically significant.

## Results

In total, 130 (60.7%) of the 214 patients hospitalized in the palliative care unit during the study period were included in the analysis. Of the 84 excluded patients, 75 (89.3%) were unable to complete the questionnaires (33 with worsening physical health, 29 with clouding of consciousness such as somnolence, 9 with neurocognitive disorders, and 4 with anxiety neurosis). In addition, 6 patients refused to participate, and 3 patients were hospitalized for only a short time, such as 2 days. Patients’ characteristics are summarized in Table 1. Patients’ median age was 74 years, and the lung was the most common primary cancer site (*n* = 31, 23.8%). The total number of metastases was highest in the liver (*n* = 54, 41.5%), followed by the lung (*n* = 52, 40.0%). At the end of the study period, 109 patients were confirmed dead, and information was missing for 18 and 3 patients because they left the hospital before death and remained hospitalized, respectively. Survival times were < 3 weeks for > 50% of patients (61/109; 56.0%). Table 1 also shows the baseline QLQ-C15-PAL scores and inflammatory biomarkers at the time of hospitalization. Regarding the symptom scales, the highest median scores were observed for fatigue (66.7) and appetite loss (66.7). Most patients showed abnormally high CRP (> 1.0 mg/dL, 105/126; 83.3%), abnormally low Alb (< 3.5 g/dL, 115/126; 91.3%), and an mGPS of 2 (98/126; 77.8%), indicating that the study population showed increased inflammation.

**Table 1:** Characteristics of patients in the study population

	Number (%)
Total Number of Patients	130
Age, years (median, range)	74 (32–97)
Sex (Male/Female)	71 (54.6)/59 (45.4)
Primary Cancer Site	
Lung	31 (23.8)
Colon	26 (20.0)
Pancreas	12 (9.2)
Liver	7 (5.4)
Stomach	7 (5.4)
Esophagus	5 (3.8)
Breast	5 (3.8)
Ovary	4 (3.1)
Uterine	4 (3.1)
Prostate	3 (2.3)
Other	23 (17.7)
Unknown	3 (2.3)
Metastasis (total number)	
Liver	54 (41.5)
Lung	52 (40.0)
Bone	40 (30.8)
Brain	14 (10.8)
PPS	
≥ 70	20 (15.4)
40–60	74 (56.9)
≤ 30	29 (22.3)
Unknown	7 (5.4)
Survival, days (median, range) *n* = 109	18 (2–193)
QLQ-C15-PAL (median (Q1, Q3), mean (± SD))	
Physical Functioning (*n* = 125)	33.3 (20.0, 46.7), 37.0 ± 25.1
Emotional Functioning (*n* = 129)	66.7 (41.7, 83.3), 63.2 ± 28.3
Dyspnea	33.3 (0.0, 66.7), 39.5 ± 34.4
Pain (n = 129)	50.0 (16.7, 66.7), 47.3 ± 34.0
Insomnia	33.3 (0.0, 66.7), 40.0 ± 36.0
Appetite Loss	66.7 (33.3, 100), 57.7 ± 38.4
Constipation (*n* = 128)	33.3 (0.0, 66.7), 37.0 ± 35.3
Fatigue (n = 129)	66.7 (44.4, 100), 63.8 ± 29.7
Nausea/Vomiting	0.0 (0.0, 16.7), 18.7 ± 29.6
QOL (*n* = 124)	50.0 (16.7, 50.0), 38.7 ± 27.1
Inflammatory Biomarkers (median, range) *n* = 126	
CRP (mg/dL)	3.9 (< 0.1–32.1)
Alb (g/dL)	2.6 (1.2–3.9)
NLR (*n* = 122)	7.6 (0.6–187)

*Alb* albumin, *CRP* C-reactive protein, *NLR* neutrophil-lymphocyte ratio, *PPS* Palliative Performance Scale, *QLQ-C15-PAL* European Organization for Research and Treatment of Cancer Quality of Life Questionnaire Core 15 Palliative Care, *QOL* quality of life

In the QLQ-C15-PAL, patients rated their symptoms using a 4-point scale (1 = not at all, 2 = a little, 3 = quite a bit, 4 = very much) for two functional domains and 7 symptom domains, and a 7-point scale (range: 1 = very poor to 7 = excellent) for overall QOL. All scale scores were linearly transformed, and the resultant scores ranged from 0 to 100. Q1: first quarter (25% quartile), Q3: third quarter (75% quartile), SD: standard deviation

Table 2 shows the results of the Cox proportional hazard model analysis of the dependence of survival times on QLQ-C15-PAL scores and inflammatory biomarkers. In the univariate analysis, statistically significant predictors of survival were the QLQ-C15-PAL dyspnea and fatigue scores and levels of the inflammatory biomarkers CRP, Alb, and NLR. Among the QLQ-C15-PAL scales, higher scores of dyspnea (hazard ratio, HR = 1.010) and fatigue (HR = 1.011) were significantly associated with shorter survival. Additionally, higher CRP (HR = 1.050) and NLR (HR = 1.017) or lower Alb (HR = 0.628) were significantly associated with shorter survival. A multivariate analysis showed that the dyspnea and fatigue scores and Alb and NLR levels were significantly related to survival.

**Table 2:** Results of Cox proportional hazard model analysis of survival data

	Univariate analysis						Multivariate analysis (*n* = 112)				
variables	n	estimates	S.E.	HR	95% CI	p	estimates	S.E.	HR	95% CI	p
QLQ-C15-PAL											
Physical Functioning	125	−0.001	0.004	0.999	0.991–1.006	.738					
Emotional Functioning	129	0.001	0.003	1.001	0.994–1.008	.809					
Dyspnea	130	0.010	0.003	1.010	1.005–1.016	< .001**	0.012	0.003	1.012	1.005–1.019	< .001**
Pain	129	−0.0004	0.003	1.000	0.994–1.005	.878					
Insomnia	130	0.001	0.003	1.001	0.996–1.006	.608					
Appetite Loss	130	0.005	0.003	1.005	1.000–1.010	.073					
Constipation	128	0.0003	0.003	1.000	0.995–1.006	.922					
Fatigue	129	0.011	0.003	1.011	1.004–1.017	.002**	0.009	0.004	1.009	1.001–1.016	.024*
Nausea/Vomiting	130	0.002	0.003	1.002	0.995–1.008	.597					
QOL	124	−0.007	0.004	0.993	0.986–1.000	.055					
Inflammatory Biomarkers											
CRP	126	0.049	0.014	1.050	1.022–1.079	< .001**					
Alb	126	−0.465	0.171	0.628	0.449–0.878	.007**	−0.457	0.180	0.633	0.445–0.902	.011*
NLR	122	0.017	0.003	1.017	1.012–1.023	< .001**	0.015	0.003	1.015	1.009–1.021	< .001**

*p < .05, **p < .01; estimates = regression coefficients, S.E.: Standard errors of estimates; all QLQ-C15-PAL scale scores were linearly transformed, and the resultant scores ranged from 0 to 100. *Alb* albumin, *CI* confidence interval, *CRP* C-reactive protein, *HR* hazard ratio, *NLR* neutrophil-lymphocyte ratio, *QLQ-C15-PAL* European Organization for Research and Treatment of Cancer Quality of Life Questionnaire Core 15 Palliative Care, *QOL* quality of life

**Fig. 1:**
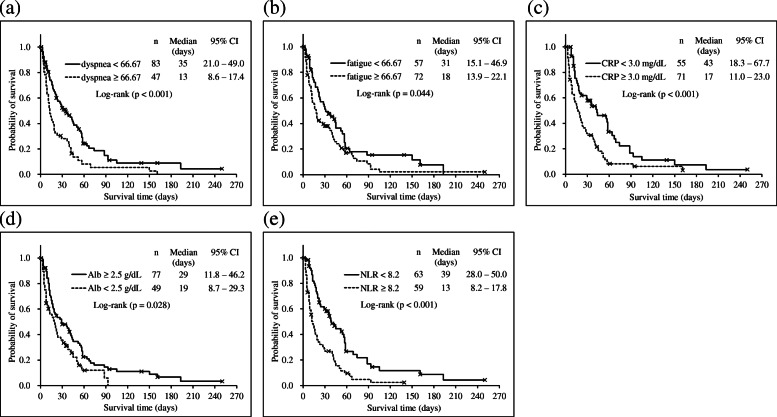
Survival curves of the categorized groups according to the cut-off values for **a** dyspnea and **b** fatigue QLQ-C15-PAL scores, **c** C-reactive protein (CRP), **d** albumin (Alb), **e** neutrophil-lymphocyte ratio (NLR). The inserted numbers represent the number of patients (n), median of the survival day in each group, and a 95% confidence interval (CI)

Results of the ROC analysis of the statistically significant variables to detect prognostic risk of < 3 weeks are summarized in Table 3. The cut-off values were estimated at 66.67 (dyspnea), 66.67 (fatigue), 3.0 mg/dL (CRP), 2.5 g/dL (Alb), and 8.2 (NLR). A transformed score of > 66.67 corresponds to the sum of raw scores (Q7 and Q11) of > 6 for fatigue and > 3 for dyspnea. The areas under ROC curves (AUCs) were 0.6–0.7 and statistically significant in all of those indicators except for Alb (*p* = .06), and both sensitivity and specificity were 0.5–0.7. Figure 1 shows the Kaplan-Meier curves of these indicators for the groups categorized using each of the cut-off values, and the log-rank test indicated significant differences between groups (*p* < .05 for all cases).

**Table 3:** Cut-off values of each indicator for detecting risk of a prognosis of < 3 weeks

Indicators	n ^a^	Cut-off	Sensitivity	Specificity	AUC	95% CI	p ^b^
Dyspnea	109	66.67	0.508	0.729	0.661	0.560–0.761	.002**
Fatigue	108	66.67	0.683	0.542	0.691	0.593–0.789	< .001**
CRP (mg/dL)	106	3.0	0.678	0.489	0.612	0.504–0.720	.043*
Alb (g/dL)	106	2.5	0.593	0.638	0.604	0.496–0.713	.060
NLR	102	8.2	0.667	0.644	0.699	0.598–0.801	< .001**

*p < .05, **p < .01; ^a^Number of patients whose survival time data were available. ^b^*p* value derived according to the receiver operating characteristic curve analysis. All QLQ-C15-PAL scale scores were linearly transformed, and the resultant scores ranged from 0 to 100. *Alb* albumin, *AUC* area under the curve, *CI* confidence interval, *CRP* C-reactive protein, *NLR* neutrophil-lymphocyte ratio, *QLQ-C15-PAL* European Organization for Research and Treatment of Cancer Quality of Life Questionnaire Core 15 Palliative Care

## Discussion

We performed an observational study to examine the role of QLQ-C15-PAL scores and the inflammatory biomarkers CRP, Alb, and NLR for survival prediction in terminally ill patients with cancer hospitalized in a palliative care unit. Our study identified that dyspnea and fatigue symptom scores in the QLQ-C15-PAL and all inflammatory biomarkers could be independent prognostic factors of short-term survival for terminally ill patients with cancer. We further revealed the cut-off values for each of the dyspnea and fatigue symptom scores for predicting prognosis.

Although previous studies [7, 9, 10] have reported that several scores in the QLQ-C30 were associated with survival, there are few reports regarding the QLQ-C15-PAL. Lee et al. [8] reported that multiple QLQ-C15-PAL scores can be independent prognostic factors of survival in patients with far advanced cancer; however, our results indicate only dyspnea and fatigue symptoms as the independent prognostic factors in QLQ-C15-PAL scores. Our study included patients with terminally ill cancer, many of whom had low PPS (≤ 60, 103/130; 79.2%) and short survival times of 18 days (median). These results suggest that the dyspnea and fatigue symptoms among QLQ-C15-PAL scores are independent prognostic factors, particularly in terminally ill patients with cancer.

Many previous studies have identified PROs as prognostic factors for survival of patients with cancer [7–11], but few have examined the cut-off values for PRO measurements to predict survival. Therefore, we examined the cut-off values of dyspnea and fatigue in the QLQ-C15-PAL for detecting the risk of a short-term prognosis of < 3 weeks (21 days). Both dyspnea and fatigue showed cut-off values of 66.67 (transformed QLQ-C15-PAL scores). However, severe fatigue was indicated with transformed QLQ-C30 scores of ≥66.67 in a previous study [32]. Meanwhile, in our study, more than 50% of patients showed severe fatigue according to the QLQ-C15-PAL scores. These results suggest that the cut-off values of transformed QLQ-C15-PAL scores, which can lead to severe levels of dyspnea and fatigue symptoms experienced by many terminally ill patients, are a predictive indicator of short-term prognosis.

The levels of CRP, Alb, and NLR are known prognostic indicators for patients with cancer [18–24], which was confirmed in the Cox analysis (Table 2). We also identified cut-off values for these markers to predict 3-week survival (Table 3). Considering the criteria for an mGPS of 2 (CRP > 1.0 mg/dL and Alb < 3.5 g/dL), our results indicate that a high cut-off value for CRP (3.0 mg/dL) and a low cut-off value for Alb (2.5 g/dL) are estimates from advanced cancer patients near the end of life. Additionally, approximately 80% of patients had a CRP > 1.0 mg/dL, and over 70% had an mGPS of 2 in our study. This is consistent with previous reports in palliative care settings [18, 33]; therefore, terminally ill cancer patients might be at greater risk for chronic inflammation. Moreover, in cancer cachexia, inflammatory cytokines, such as IL-6 derived from cancer cells, act on hepatocytes to increase CRP production and increase protein catabolism [17]. The level of Alb, reflecting the visceral protein pool [34], is reduced in the presence of a systemic inflammatory response [34, 35].

NLRs have been reported as inflammatory biomarkers, based on neutrophil and lymphocyte counts [29]. A systematic review indicated that an NLR score of 4 was the best cut-off value for prognosis [24]. On the other hand, our study revealed that the cut-off value for the NLR was 8.2, and most patients had abnormally high NLR scores (> 4, 98/122; 80.3%). This is a similar finding to a previous study that reported scores of 9.21 in cancer patients who died within 4 weeks [23]. These findings also suggest increased inflammation in terminally ill patients with cancer. Although there are many biomarkers that can be influenced by systemic inflammatory responses, in our study, we chose only CRP, Alb, and NLR measurements because of strong evidence for their association with prognoses. In addition, it was impossible to measure all biomarkers related to inflammation. Therefore, we adopted the results of a univariate analysis and calculated cut-off values of each indicator. The current results suggest that the assessment of the systemic inflammatory response using these biomarkers is also useful in estimating prognoses for terminally ill patients with cancer.

Among the significant prognostic indicators, the sensitivity and specificity of patient-reported dyspnea and fatigue were comparable to that of the inflammatory biomarkers CRP, Alb, and NLR (Table 3). The measurements of PROs can be assessed directly by the patient themselves, even if no patients’ blood data were available. Near the end of life, the clinical prediction of survival requires simple, always-available prognostic indicators because the patient’s general condition rapidly deteriorates in the last month before death [3]. Therefore, the PRO score might be a more suitable prognostic indicator for short-term prognosis of 3 weeks as compared to laboratory values. The results of the present study suggest that monitoring QLQ-C15-PAL scores is necessary, along with measuring inflammatory biomarkers, in terminally ill patients with cancer.

This study had some limitations. First, it was conducted in a single hospital, and the sample size was not large enough to ensure generalizability. Second, we could not evaluate the details of various factors that could affect inflammation status, such as use of concomitant drugs like anti-inflammatory medication, nutritional intake, cancer type, and coexisting infections. Third, we could not evaluate the confounding factors likely to be associated with survival such as classification of cancer cachexia, cancer stage, age, other comorbidities. Finally, patients who were unable to answer the questionnaire were excluded, and thus, our finding might be different in patients with cognitive impairments or lacking consciousness.

## Conclusion

We examined the survival predictability of QLQ-C15-PAL scores and inflammatory biomarkers in terminally ill patients with cancer and found that the QLQ-C15-PAL dyspnea and fatigue scores could be independent prognostic indicators comparable to the inflammatory biomarkers CRP, Alb, and NLR. The findings suggest that the assessment of not only objective indicators for the systemic inflammatory response but also PROs is useful for the prediction of short-term survival in terminally ill patients with cancer.

## Supplementary Information

**Additional file 1.**

## Data Availability

The datasets used and analyzed during the current study are available from the corresponding author on reasonable request.

## Abbreviations

EORTC: European Organization for Research and Treatment of Cancer
QLQ-C15-PAL: Quality of Life Questionnaire Core 15 Palliative
CRP: C-Reactive Protein
Alb: Albumin
NLR: Neutrophil-Lymphocyte Ratio
ROC: Receiver Operating Characteristic
PRO: Patient-Reported Outcome
QOL: Quality of Life
QLQ-C30: Quality of Life Questionnaire Core 30
mGPS: modified Glasgow Prognostic Score
PPS: Palliative Performance Scale
HR: Hazard Ratio
AUC: Areas Under ROC Curve
